# Cultivation and Nutritional Evaluation of *Agaricus bisporus* with Tea Residue as Culture Medium

**DOI:** 10.3390/foods12132440

**Published:** 2023-06-21

**Authors:** Zhuochen Wang, Mengru Li, Jundi Fan, Yuting Bao, Qi Chen

**Affiliations:** 1 Institute of Agro-Products Processing, Anhui Academy of Agricultural Science, Hefei 230031, China; wzc45098@163.com (Z.W.);; 2 Anhui Engineering Laboratory of Food Microbial Fermentation and Functional Application, Hefei 230031, China; 3 State Key Laboratory of Tea Plant Biology and Utilization, Anhui Agricultural University, Hefei 230036, China; 4 Anhui Engineering Laboratory for Agro-Products Processing, School of Tea and Food Science & Technology, Anhui Agricultural University, Hefei 230036, China

**Keywords:** button mushroom (*Agaricus bisporus*), cultivation substrate, tea extraction residue, flavor compounds, gas chromatography-mass spectrometry (GC-MS)

## Abstract

Different constituents of the cultivation substrate have significant effect on the yield and quality of edible mushrooms. The residue after the extraction of instant tea has exhibited to be biologically significant, and could be used as a substrate for cultivation. This study aimed to investigate the feasibility of tea extraction residue (TER) on button mushroom (*Agaricus bisporus*) cultivation, as an ingredient in the substrate, and assess the growth status, nutritional values, and sensory characteristics of fruiting body. The results showed that the strains could grow well on the cultivated substrate with 20% addition of TER. The total amount of hydrolyzed amino acids in the fruiting bodies of three TER-based groups (TER accounted for 10%, 20%, and 37.5%, respectively) was higher than that of the control group, and the total amount of essential amino acids was increased by 33.33%, 22.47%, and 9.92% compared with the control group, respectively. In addition, the results of gas chromatography-mass spectrometry (GC-MS) revealed that the addition of TER to the cultivation of substrate significantly enhanced the content of typical mushroom-flavor compounds in button mushroom, such as 1-octen-3-ol, 3-octanol, and 1-octen-3-one. It can be concluded that TER may be an ideal choice for the substrate in commercial cultivation of button mushroom.

## 1. Introduction

Since the late 1990s, the global yield of edible mushrooms has increased significantly due to appropriate cultivation conditions and awareness of their high nutritional value. Among them, button mushroom (*Agaricus bisporus*) was widely cultivated and consumed worldwide, accounting for 15% of the global mushroom production [1]. At present, the commercial substrate for cultivation is mainly consisted of agricultural residues wheat straw, poultry manure, nitrogen-containing additives (soybean meal or synthetic nitrogen, including urea or ammonium nitrate), and gypsum under appropriate conditions by microbial fermentation [2]. The substrate compositions exhibited to have significant effects on growth, chemical and nutritional compositions, influencing the growth of mycelium, characteristics of fruiting body, and nutritional value of cultivated mushrooms [3,4,5,6]. Therefore, optimal substrate formulation is of great significance to improve the nutritional value of button mushroom.

Instant tea is a popular beverage worldwide, and has been favored by consumers for its convenience, unique flavor, and nutritional value [7]. The annual production capacity of instant tea and concentrated tea in China was nearly 20,000 t [8]. Therefore, remarkable amounts of tea extraction residues (TER) were annually produced in the processing of instant tea. Although they are deposited as an agricultural waste, they still contained several nutrients, about 4.31% and 50.3% of carbon and nitrogen respectively [9]. As reported previously, TER contains 1–2% tea polyphenol, 0.1–0.3% theanine, 16–18% fiber, and 17–19% crude protein, with a high potential of utilization [10]. Peksen and Yakupoglu (2009) [11] reported that a certain proportion of TER could serve as the main source of energy and nutrient for cultivation substrate of *Ganoderma lucidum*, while significantly increase the yield and bioavailability of fruit body. In the study of Gülser and Pekşen (2003) [12], a mixture of TER with peat in a ratio of 1:1 (*v*/*v*) could be used profitably as a new mulch soil in cultivation of button mushroom. Experiments by Atİla (2019) [13] and Niazi et al. (2022) [14] confirmed that TER was economical and effective as the growing medium for the cultivation of *Lentinus tigrinus* or *Lentinula edodes*. Although TER can be used as substrate for cultivation of mushrooms, their effects on nutritional and organoleptic properties of button mushroom cultivated by TER, have still remained elusive. The present study aims to investigate the optimal cultivation composition and formulas based on raw materials, including TER, poultry manure, and wheat straw. The cultivated button mushroom was comparatively evaluated regarding mycelium growth, yield, content of polysaccharides, crude proteins, and free amino acids. Furthermore, the volatile flavor compounds of fruiting body were compared as well. Our results may provide a novel idea for recycling the agricultural residues.

## 2. Materials and Methods

### 2.1. Substrate Materials

Soybean meal, chicken manure, and wheat straw used as substrate materials for cultivation of button mushroom were provided by Duoduoli Agricultural Science and Technology Co., Ltd. (Fuyang, Anhui province, China). TER were purchased from a tea company located in Huangshan, Anhui province, China.

### 2.2. Chemicals

All solvents used for sample preparation were of high-performance liquid chromatography (HPLC) grade. Amino acid derivatization was achieved with AccQ-Fluor reagent Kit (Waters Corp., Milford, MA, USA). All solvents used for sample analysis, methanol (MeOH), and acetonitrile (ACN), were of liquid chromatography-mass spectrometry (LC-MS) grade and purchased from Tedia Company Inc. (Fairfield, OH, USA). Calcium chloride and ethyl decanoate (internal standard) were purchased from Aladdin Company Inc. (Shanghai, China). C7–C25 saturated alkanes were provided by Sigma-Aldrich (St. Louis, MO, USA) and used for calculating retention indices. H_2_O was purified on a Milli-Q system (Merck Millipore, Billerica, MA, USA).

### 2.3. Compost Formulation

The substrate formula of *Agaricus bisporus* used by the cooperative plantation was composed of wheat straw, chicken manure, soybean meal, gypsum, and lime in the radio of 70:18:10:1:1 (on dry weight basis), which was used as control group. An automatic elemental analyzer (Vario EL cube; Elementar Analysensysteme GmbH, Langenselbold, Germany) was applied to determine the content of carbon and nitrogen in the dried wheat straw, chicken manure, soybean meal, and TER. Soybean meal was removed, the content of other components was adjusted according to the carbon and nitrogen content test results, and three new culture formulas (T1, T2, and T3) were formulated using TER, accounting for 10%, 20%, and 37.5%, respectively (The specific formulas used in the present study are listed in Table S1).

### 2.4. Cultivation of Button Mushroom

After mixing materials with the above-mentioned proportion, the standard industrialized cultivation process of button mushroom was carried out in accordance with pile fermentation, maturity fermentation, inoculum, spawn run, casing, fruiting and harvesting [15]. The period from inoculation to the first harvest was observed and recorded (five flushes of mushroom were harvested). The cultivation area of button mushroom was 15 m^2^ under each substrate formula, and it was repeated three times. The first flush of button mushrooms was collected to remove the stipe and clean. Some fresh mushrooms were selected to immediately measure the whiteness and hardness. Some mushroom samples were stored in a −80 °C refrigerator for determination of nutritional value, and another part was freeze-dried for subsequent detection of volatile flavor compounds.

Typical agronomic and quality traits of *Agaricus bisporus* include earliness (first harvesting day), yield, growth rate, maturation, size, color, shape, firmness, aroma, and shelf life [16]. In the current study, the growth-based parameters (mycelium growth, earliness and yield), the quality traits of fruiting body (whiteness, firmness), contents of nutrients (polysaccharides, crude proteins and amino acids) and volatile flavor compounds were determined.

### 2.5. Color and Firmness Assay

The surface color of mushroom caps was detected by using a colorimeter (Chroma Meter CR-400; Konica Minolta Sensing, Inc., Ramsey, NJ, USA). L* (light/dark), a* (red/green), and b* (yellow/blue) values were used to calculate the whiteness index (WI) of button mushroom. The WI was calculated from the following equation: WI = L + 3a − 3b [17].

The button mushroom samples that have been tested for WI were used to measure the hardness of the caps using a texture analyzer [18].

### 2.6. Analysis of Crude Proteins and Polysaccharides

The crude protein content of fresh button mushroom was determined by KJELDAHL procedure according the China National Standard for Determination of Protein in Food [19].

Different dried samples were ground finely. The powders (0.2 g) were added to 15 mL 85% ethanol, then extracted in 60 °C water bath for 30 min, and filtration was repeated for three times to make up to 200 mL. Using glucose as a standard, a linear regression equation of glucose was formulated by the phenol-sulfuric acid method [20] to determine the contents of polysaccharides in button mushroom.

### 2.7. Analysis of Free Amino Acids

The button mushroom samples (in form of freeze-dried power) were accurately weighed at 0.05 g and transferred to a 15 mL tube, then 5 mL 80% ethanol was added, and the samples were extracted at 50 °C in a water bath for 30 min before centrifugation. The contents of amino acids were measured by reversed-phase HPLC (RD-HPLC) with AccQ-Tag pre-column derivatization method. The analysis was carried out using a Waters 2695 HPLC System (Waters Corp., Milford, MA, USA) and fitted with a reverse-phase C18 column (5 μm, 250 mm × 4.6, Phenomenex, Torrance, CA, USA). Besides, 10%phosphate-buffered saline (PBS), acetonitrile, and ultra-pure water were used as mobile phases A, B, and C, respectively, for gradient elution, and the injection volume was 10 μL. The gradient elution program was optimized as follows: 5% B and 4% C for 0 ~17 min; 17% B and 3% C for 24 min; 20% B and 12% C for 32 min; 40% B and 60% C for 35 min; 60% B and 40% C for 37 min; 0% B and 0% C for 38 min, keeping to 45 min. The flow rate was 1 mL/min, and the column temperature was 37 °C. The content of amino acids in different samples was calculated according to calibration curve equation.

### 2.8. Analysis of Flavor Compounds

Extraction of Volatiles. Flavor volatiles were measured according to the method presented by [21] with modifications. The freeze-dried button mushroom powders were accurately weighed at 0.5 g, and then mixed with 2.5 mL CaCl_2_ and 50 μL internal standard (5 mg/L ethyl decanoate in methyl alcohol, *v/v*). The mixture was hermetically sealed in a 15 mL vial and magnetically stirred. The solid phase microextraction (SPME) technique was employed using fused-silica fibers coated with a 50/30 μm layer of Divinylbenzene/Carboxen/Polydimethylsiloxane (DVB/CAR/PDMS) (Supelco Inc., Bellefonte, PN, USA). Then, the fibers were exposed to the headspace with maintaining the sample at 60 °C for 30 min. Finally, the fibers were inserted into the injection port of the gas chromatograph.

GC-MS analysis. GC-MS analysis was undertaken using an Agilent 7890A gas chromatograph-5975C mass selective detector equipped with a DB-5MS column (30 m × 0.25 mm × 0.25 μm, Agilent Technologies Inc., Santa Clara, CA, USA). The carrier gas was helium at a constant flow rate of 1.0 mL/min, with splitless mode. The temperature of oven was set to 50 °C for 1 min, increased to 100 °C at 2 °C/min, and then elevated to 280 °C at 10 °C/min, and held for 10 min. The temperature of injector was set to 250 °C. The mass detector was operated in the electron impact mode with an ionization energy of 70 eV and a scanning range of 30–550 amu.

Identification of Volatiles. The volatiles were positively identified by comparing mass spectra and retention indices (RIs). RIs of the volatiles were calculated according to the retention time of mixture components and normal alkanes (C_7_–C_25_). The semiquantitative analysis of volatiles was performed by comparing their peak areas with that of the internal standard compound on the GC-MS total ion chromatogram.

### 2.9. Statistical Analysis

Data were expressed as the mean ± standard deviation (SD) of three replications. One-way analysis of variance (ANOVA) was performed by using SPSS 20.0 software (IBM, Armonk, NY, USA), and Duncan’s new multiple range test was applied to compare the significant difference (*p* < 0.05). The experimental data from the SPME-GC-MS were analyzed by SIMCA 14.1 software (Umetrics, Umeå, Sweden).

## 3. Results and Discussion

### 3.1. Dynamic Changes of Substrate Components before and after Fermentation

The contents of carbon and nitrogen in TER, chicken manure, straw, and soybean meal were detected by an automatic element analyzer (Table S1), and the results showed that the mass percentage of carbon and nitrogen elements in TER was 50.40% and 5.91%, respectively, which was higher than that of soybean meal (45.20%, 4.54%). Soybean meal is commonly used to provide nitrogen source, however, due to its tight supply and demand and rising prices in China, TER were alternatively utilized in the present study. 

Table S2 shows that the total amount of C and N was reduced by pile and maturity fermentation, which was caused by the large amount of ammonia and carbon dioxide produced during the decomposition process. It was revealed that in TER-based groups, the retention rate of element C was relatively consistent, while that of element N was quite different. This indicated that with the increase of TER, the nitrogen content gradually raised, and the release of ammonia from decomposition of microorganism compounds accordingly enhanced, leading to more nitrogen depletion, which was the main reason for the increase of C/N ratio in the substrate after fermentation [2,22]. Thus, it can be concluded that in the later stage of fermentation, the number of turning over and ventilation should be appropriately increased to promote the emission of NH_3_ and N_2_O, as well as fully decomposition of high TER substrate.

### 3.2. The Growth Status

As presented in Table 1, there was a significant difference in growth status between the control group and the TER group. A study by Yang et al. (2016) [23] found that the addition appropriate ratios tea residue could be used as an effective and economic substrate for oyster mushroom cultivation resulted in high fruiting body yield, high biological efficiency, and relatively short planting time, which had similar results to the present study. It showed that addition of a certain proportion of TER into the substrate had a positive effect on promoting mycelium growth, shortening earliness, and increasing yield. That may be related to the change of physical properties of the substrate. Adding tea residue can improve the water retention and air permeability of the medium, which is conducive to the growth of the mycelium of tricholoma dicporus [24]. However, it was observed that excessively high proportion of TER would lead to negative consequences. The cultivation of substrates containing 100% TER (the C/N ratio after fermentation was 15.70) was carried out in the early stage, and the results unveiled that utilization of TER as the sole source of nutrients could lead to the sharply growth of mycelium. According to the growth status of T3 group, the growth of mycelium and the yield of button mushroom were the lowest in the treatment group. It indicated that the C/N ratio of substrate plays a leading role in mycelium growth, as well as fruiting body formation and development [25]. According to a previous experience, when the C/N ratio of *A. bisporus* cultivation substrate is about 18~20: 1, the mycelium grows better and the yield is higher. The excessive addition of TER may reduce the C/N ratio of the substrate, resulting in the scarcity of available carbon source to making the formation of carbon skeleton, thereby influencing the mycelium growth rate and yield of button mushroom. In the study of Rawiningtyas et al. (2023) [26], high reed content in the substrate with low C/N ratio led to a decrease in the yield of wood ear mushroom, which had similar results to our study. Carbon source could support the growth and development of mushrooms, while low C/N ratio would lead to lack of carbon source in the medium, resulting in the reduction of yield. Moreover, the result of the growth status of T3 group may also be due to the high content of polyphenols in TER, which inhibits the growth of mycelia and affects the yield of mushrooms [5,27].

### 3.3. Assessment of Color and Firmness

The whiteness and hardness of button mushroom are important parameters affecting its quality and a consumer’s acceptance [18]. In the current study, the button mushroom in TER group showed higher whiteness value than control group (51.06 (control group) vs. 60.40 (T2 group)), which presented an acceptable quality. At the same time, the hardness of mushroom declined with increased rate of TER in the cultivation process. For whiteness and hardness, there was a significant difference between the TER group and the control group only when the amount of TER was more than 10% in substrate (*p* < 0.05, Table 1). A number of studies demonstrated that the whiteness and firmness of mushrooms have been influenced by the nature of the substrates employed in mushroom cultivation. For instance, button mushrooms growing in quail manure were whiter (showed higher L value) compared with chicken manure when they were freshly picked [28]. The hardness of *Pleurotus florida* by cultivated with cotton seed and rice stalk was reduced by 45%, and the texture became softer, which was mainly caused by the reduction of total dietary fiber [29]. This was consistent with previous data that excessive addition of TER could influence the skeleton formation of *A. bisporus*, thereby affecting the hardness of fruiting bodies.

### 3.4. Analysis of Crude Proteins and Polysaccharides

Mushrooms are globally appreciated for their nutritional value and medicinal properties [30]. Moreover, polysaccharides are compounds with important medicinal bioactivity in button mushroom with antioxidant activities and anti-aging effects [31].

In the present research, the crude protein content of button mushroom varied between 2.70~2.85 g/100 g, and T1 group exhibited the greatest effect. The content of polysaccharides in the control group was 13.07 g/100 g, and that was 12.49, 13.06, and 12.73 g/100 g in the TER-based (T1, T2, T3) groups, respectively (Table 1). The substrate with different TER ratios had no significant influence on the formation of primary metabolites of button mushroom, which was in agreement with the finding from Altieri et al. (2009) [32]. In their study, low differences were observed in the total proteins and total carbohydrates of button mushroom grown on an experimental substrate containing olive mill waste and control substrates. In the study of Yang et al. (2014) [33], it was found that there was a negative correlation between the polysaccharide content of oyster mushroom planted in tea residue medium and the proportion of tea residue added, which was consistent with our observation.

### 3.5. Analysis of Free Amino Acids

Amino acid has various physiological functions and is an important substance to support life activities. Many studies demonstrated that the type of compost did not influence the essential amino-acid composition, and cups contained more protein than stipes in all stages of development of mushrooms [34]. As shown in Table 2, the type of hydrolyzed amino acids in the fruiting body was complete. Compared with the control group, the total amount of essential amino acids in the TER-based groups (T1, T2, T3) was increased by 33.33%, 22.47%, and 9.92%, and the total amino acid content was elevated by 13.16%, 7.12%, and 7.01%, respectively. These results revealed that the amount of total amino acids was significantly increased by the moderate addition of TER, which was consistent with the observation of the growth state. Meanwhile, the increase of essential amino acids was more obvious, which was in accordance with Shashirekha et al.’s findings (2005) [29]. Yang et al. (2014) [33] also observed that the mushroom fruity body cultivated in tea residue medium with more nutritious, and the total amino acid and essential amino acid content in the mushroom fruity body increased with the increase of the tea residue proportion in substance.

Fujihara et al. (2000) [35] pointed out that content of amino acids in the fruiting bodies of shiitake was closely associated with the content of nitrogen in the sawdust medium. In the present experiment, the content of nitrogen in the compost increased with the addition of TER (Table S2), which provided a rich material basis for the formation of amino acids in the fruit bodies, whereas the content of amino acids in T3 group decreased instead, which may be due to the influence of excessive amount of TER on the C/N balance during the mycelium development. Futhermore, the amino acid content of substrate also had a great influence on the formation of amino acids in the fruiting bodies, and amino acids required for fungal metabolism could be directly absorbed from the culture substrate [5]. The residual amino acids in the TER may have promoted the formation of amino acids in the *Agaricus bisporus.*

Button mushroom is a proper source of lysine, which is a limiting amino acid in cereal-based products [34]. The lysine content in fruiting body was increased by 50% when the cultivated substrate contained 10% TER, which could improve the deficiency of lysine caused by the dietary pattern dominated by grains and cereals in China. Notably, the contents of methionine and cysteine in sulfur-containing amino acids were elevated by 275% and 433%, respectively, which could improve the nutritional value and antioxidant capacity of button mushroom.

### 3.6. Analysis of Flavor Compounds

**Figure 1:**
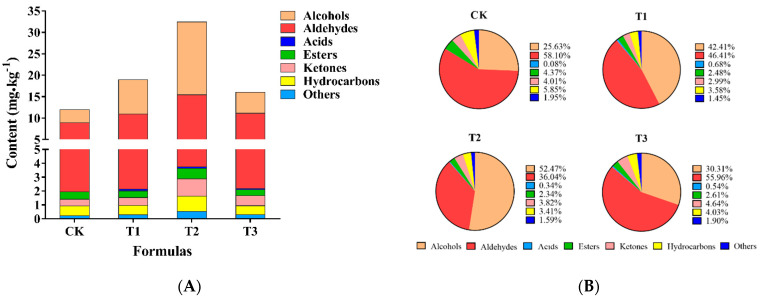
(**A**) Overall flavor compounds concentration (mg/kg button mushrooms relative to internal standard) of Agaricus bisporus cultivated in different compost. (**B**) Changes in the proportion of volatile compounds in each category under four treatments. CK, control group; T1, T2, and T3 indicate treatment groups with tea residue contents of 10%, 20%, and 37.5%, respectively.

**Figure 2:**
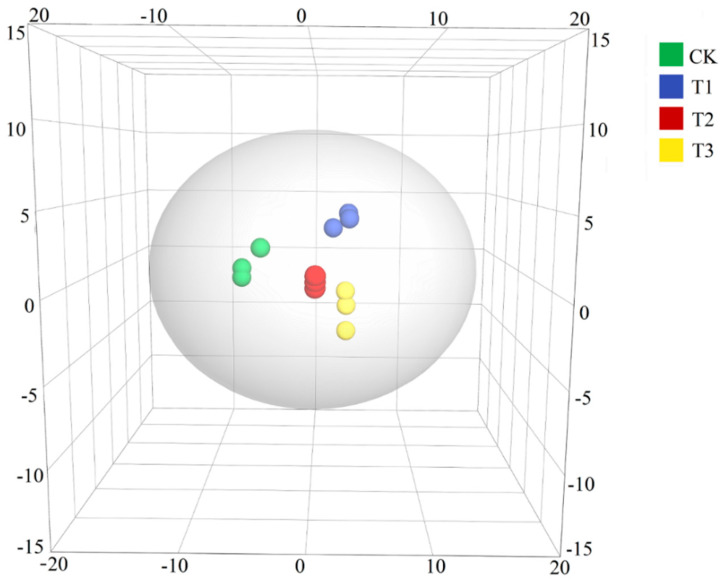
Principal component analysis (PCA) of differential volatile compounds in button mushroom samples. PC1 = 57.6%, PC2 = 22.6%, PC3 = 9.14%. CK, control group; T1, T2, and T3 indicate treatment groups with tea residue contents of 10%, 20%, and 37.5%, respectively.

**Figure 3:**
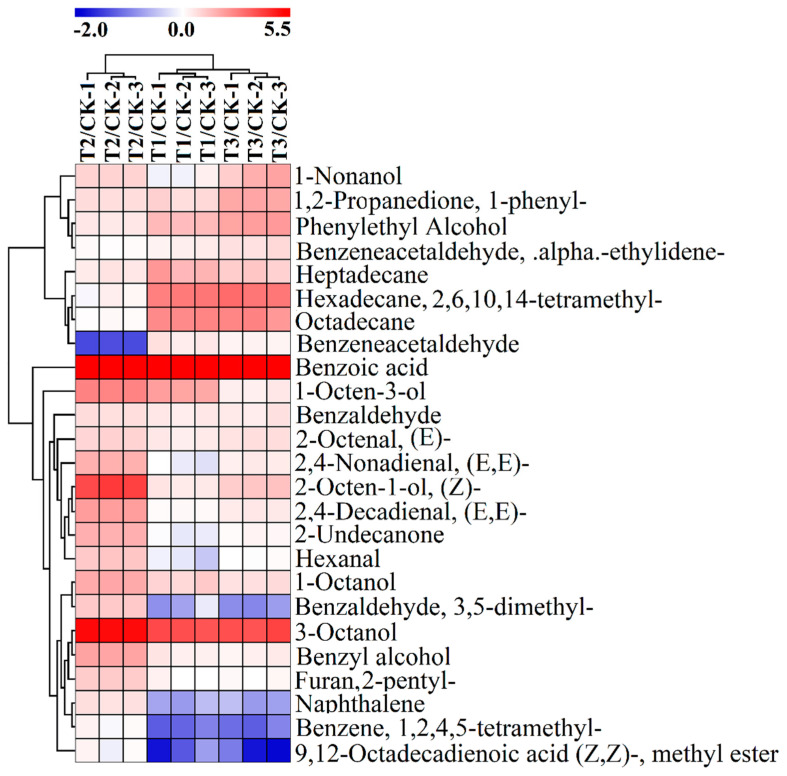
Hierarchical Clustering Analysis (HCA) of differential volatile metabolites. CK, control group; T1, T2, and T3 indicate treatment groups with tea residue contents of 10%, 20%, and 37.5%, respectively.

The highly desirable smell of mushrooms is mainly attributed to the rich volatile organic compounds (VOC_S_), and several aliphatic C_8_ compounds are the main contributors to mushroom volatiles. The profile of aroma compounds of mushroom varied with species and could also be influenced by cultivation conditions [36,37]. In the present study, the total ion chromatograph (TIC) of four different treatment groups was shown as Figure 1, total of 71, 75, 80, and 77 volatile compounds were identified in control group and TER group (T1, T2, T3), which were roughly divided into the following types, including alcohols, aldehydes, acids, esters, ketones, hydrocarbons, etc. Besides, the total amount of VOC_S_ in button mushroom cultivated in the four different substrates was 11.94, 18.95, 32.43, and 15.97 mg/kg, respectively (Figure 2). A heatmap analysis was applied to visualize the changes of these metabolites in each treatment group compared with the control group. It was uncovered that the content of the majority of compounds was increased, and the trend of changes in T1 group and T3 group was more similar. (Figure 3). The above-mentioned outcomes indicated that the aromatic compositions of button mushroom changed due to the alterations of substrate components, and an appropriate amount of TER in the substrate had a significant effect on increasing the content of volatile flavor compounds, which is likely related to the residual nutrients in the TER, promoting the synthesis of aroma compounds. Hiraide et al. (2010) [38] added amino acids to the sawdust culture medium to increase the amount of mushroom odor compounds. 

**Figure 4:**
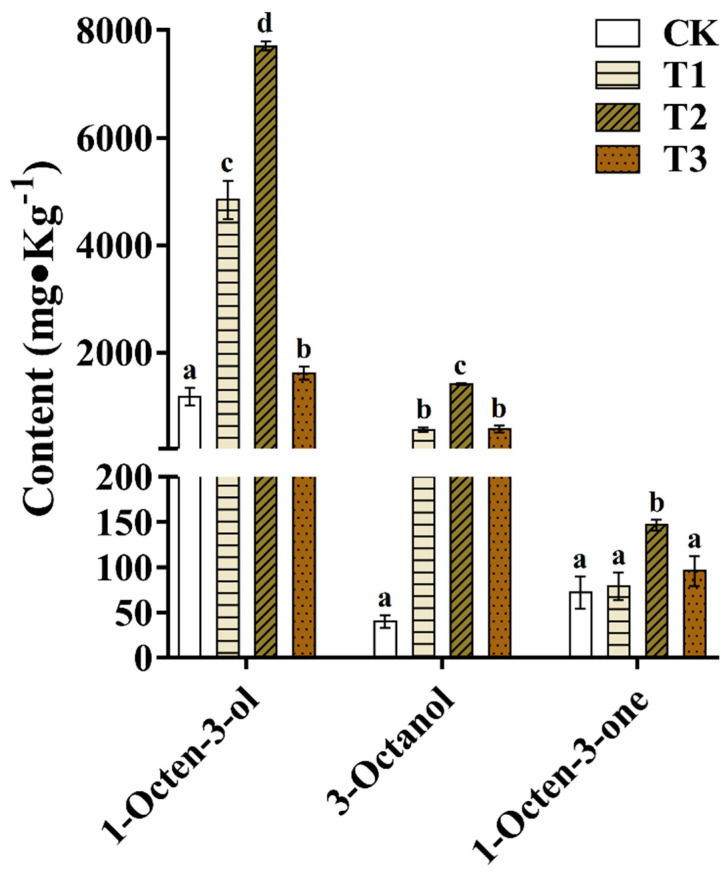
Distinctive “mushroom-like flavor” compounds concentration (mg/kg button mushrooms relative to internal standard). CK, control group; T1, T2, and T3 indicate treatment groups with tea residue contents of 10%, 20%, and 37.5%, respectively. Different superscript lowercase letters (a–d) in the columns indicate significant differences between groups (*p* < 0.05).

Principal component analysis (PCA) was applied to analyze the content of volatiles to clarify the relationship between samples and volatile components. As illustrated in Figure 4, the button mushroom cultivated in four different mediums could be easily separated in scoring plots by combining PC1 (57.6%), PC2 (22.6%), and PC3 (9.14%). In order to obtain more reliable difference metabolites information between groups, the orthogonal projections to latent structures discriminant analysis (OPLS-DA) was applied to maximumly extract information from the dataset, and to analyze the results. In this mode, the remaining variables were screened by the Student’s *t*-test (*p*-value < 0.05), and the variable importance in the projection (VIP > 0.7) of the first principal component of the OPLS-DA model was assessed [39,40,41]. A total of 25 differential metabolites were selected from 83 identified volatile compounds (Table 3). 

A variety of volatile compounds have been reported as the major aroma compounds of mushrooms, among which C8 compounds are the main source of the characteristic flavor of many mushrooms. Tagkouli (2021) [42] detected the volatiles of *Pleurotus eryngii* and *Pleurotus ostreatus* in the study, and the results showed that the volatiles were mainly C8 compounds, accounting for 78~83% and 84~91% of the total volatiles respectively, especially 1-octen-3-ol, 3-octanol, and 1-octen-3-one, which were described as distinctive “mushroom-like flavor” and considered as the main active aroma compounds, and 1-octen-3-ol was also known as “mushroom alcohol” [43,44]. As depicted in Figure 4, compared with the control group, the addition of TER into culture medium was found effective in terms of improving the amount of the three typical mushroom flavor compounds, especially in the 20% TER treatment group. These three compounds were identified as enzymic breakdown products of linoleic acids [45]. Lipoxygenase was assumed as a key enzyme for biosynthesis of the flavor compounds by forming of hydroperoxides of unsaturated fatty acids (mainly linoleic and linolenic acids), which further cleaved into volatile aldehydes and alcohols. Exogenous addition of linoleic acids to homogenate of mushroom mycelium could significantly increase the content of 1-octen-3-ol [46]. Fatty acids were reported as key precursors for aroma formation of tea, and unsaturated fatty acids were precursors of C6–C10 aroma compounds in tea [47]. Using green tea and instant green tea as the samples, the fatty acid methyl esters were prepared by the sulfuric acid-methanol method and analyzed by GC-MS, and the results showed that there were slightly free fatty acids in instant green tea [48]. Therefore, the large amount of fatty acids remaining in the TER could be beneficial to provide more flavor precursor molecules for the growth of button mushroom. This might be related to the addition of 10~20% TER to the cultured substrate, which could be advantageous to stimulate the metabolism of unsaturated fatty acids in button mushroom, thereby changing the composition and ratio of aroma compounds. Shashirekha et al. (2005) [29] demonstrated that small degrees of cotton seed powder supplementation in rice straw substrate could increase in the content of unsaturated fatty acid in *Pleurotusostreatus*, especially linolenic acids, which provided precursor molecules for the production and transformation of mushroom flavor substances. In addition, compared with the CK group, the content of benzaldehyde in the T1, T2 and T3 treatment groups all increased. This volatile compound was related to the catabolism or oxidative degradation of phenylalanine [42,49]. In the present experiment, it was observed that the content of phenylalanine in the fruite bodies produced by all tea residue treatment groups was increased, which may be the main reason for the increase of benzaldehyde content.

## 4. Conclusions

The results of the present study showed that it is feasible to replace soybean meal with agricultural waste tea residue as cultivation substrate component of *Agaricus bisporus*, and tea residue can provide rich nitrogen source and other nutrients for the growth of *A. bisporus*. It was unveiled that the optimal replacement amount of TER in the culture of button mushroom was about 20%. At this concentration, the performance of indicators, such as growth status, nutritional value, and flavor quality of button mushroom was superior than that of the control group. The content of total amino acids in fruiting body could be increased by addition of TER to the substrate, especially the essential amino acids. In addition, the total amounts of volatile compounds and some compounds with typical mushroom-like flavor, e.g., 1-octen-3-ol, 3-octanol, and 1-octen-3-one, were markedly elevated in the TER-based groups. The present research provided a novel idea for recycling the agricultural residues.

## Figures and Tables

**Table 1:** Effect of treatments on growth status, appearance and nutrient composition of fruiting body.

Treatments	Growth Status			Fruiting Body Appearance		Nutritional Composition	
	Mycelium Growth	First Harvest (Day)	Yield (Kg/m^2^)	Whiteness (WI)	Firmness (g)	Protein (g/100 g)	Polysaccharides (g/100 g)
CK	+ + +	59	21.64	51.06 ± 1.22 a	453.04 ± 3.80 a	2.82 ± 0.08 ab	13.07 ± 0.07 a
T1	+ + +	57	23.56	53.20 ± 2.58 ab	433.95 ± 3.69 ab	2.85 ± 0.04 a	12.49 ± 0.30 b
T2	+ + +	56	21.30	60.40 ± 1.81 c	411.55 ± 3.72 bc	2.70 ± 0.04 b	13.06 ± 0.21 a
T3	+ +	52	16.08	55.69 ± 1.03 b	406.63 ± 4.29 c	2.72 ± 0.05 ab	12.73 ± 0.14 ab

The results of fruiting body appearance and nutritional composition were expressed as mean values ± standard deviation. Different lowercase letters denoted a significant difference (*p* < 0.05) among the four treatments. + + + indicated that mycelium was vigorously, + + indicated that the mycelium was normal.

**Table 2:** Amino acid content of button mushroom cultivated with different composts (g/100 g).

Amino Acid	CK	T1	T2	T3
Essential amino-acid (EAA)	2.136	2.848	2.616	2.348
Val	0.24 ± 0.01 c	0.34 ± 0.01 b	0.37 ± 0.01 a	0.24 ± 0.01 c
Lys	0.42 ± 0.02 c	0.63 ± 0.01 a	0.49 ± 0.04 c	0.53 ± 0.01 b
Met	0.04 ± 0.01 e	0.15 ± 0.01 a	0.15 ± 0.01 b	0.15 ± 0.01 a
Ile	0.17 ± 0.01 c	0.18 ± 0.01 c	0.27 ± 0.03 a	0.26 ± 0.01 b
Leu	0.36 ± 0.01 c	0.42 ± 0.01 a	0.34 ± 0.06 c	0.30 ± 0.01 c
Phe	0.32 ± 0.01 c	0.56 ± 0.01 a	0.46 ± 0.01 b	0.48 ± 0.01 b
Thr	0.59 ± 0.01 b	0.58 ± 0.01 a	0.55 ± 0.01 b	0.40 ± 0.01 d
Non-essential amino acid (NEAA)	8.12	8.76	8.37	8.63
Asp	1.15 ± 0.01 a	0.74 ± 0.02 b	0.64 ± 0.01 c	0.54 ± 0.02 d
Ser	0.62 ± 0.01 d	0.95 ± 0.03 a	0.84 ± 0.02 b	0.75 ± 0.03 c
Glu	1.50 ± 0.01 a	1.29 ± 0.05 b	0.95 ± 0.03 d	1.19 ± 0.05 c
Gly	0.19 ± 0.01 c	0.39 ± 0.03 a	0.29 ± 0.05 b	0.39 ± 0.03 a
Ala	0.91 ± 0.03 a	0.80 ± 0.02 c	0.89 ± 0.03 b	0.80 ± 0.02 c
Cys	0.21 ± 0.02 a	1.12 ± 0.06 c	1.40 ± 0.02 b	1.09 ± 0.06 c
Tyr	0.25 ± 0.01 c	0.53 ± 0.01 a	0.54 ± 0.01 a	0.42 ± 0.01 b
His	1.38 ± 0.01 b	1.18 ± 0.09 c	1.65 ± 0.01 a	1.07 ± 0.09 d
Arg	1.30 ± 0.01 a	1.17 ± 0.04 b	1.17 ± 0.09 b	1.12 ± 0.09 b
Pro	0.22 ± 0.01 c	0.26 ± 0.02 b	0.31 ± 0.01 a	0.24 ± 0.05 b
TAA	10.26	11.61	10.99	10.98
EAA/NEAA	0.26	0.33	0.31	0.27
EAA/TAA	0.21	0.25	0.24	0.21

Results were expressed as means ± SD (*n* = 3). Means with different letters within a row was significantly different (*p* < 0.05). ANOVA and Duncan test were used to analyse the significant difference between samples from different substrates.

**Table 3:** Effect of Tea Residue on Volatile Flavor Compounds of button mushroom.

No.	Compounds	^b^ RI (Calculate)	RI (Reference)	^a^ Content (μg/g, Mean ± SD, *n* = 3)			
				CK	T1	T2	T3
Alcohols							
1	1-Hexanol	853	865	0.044 ± 0.009	0.062 ± 0.001	0.104 ± 0.001	0.055 ± 0.007
2	1-Octen-3-ol	974	977	1.182 ± 0.133	4.846 ± 0.29	7.709 ± 0.069	1.616 ± 0.1
3	3-Octanol	992	997	0.04 ± 0.006	0.56 ± 0.036	1.41 ± 0.013	0.576 ± 0.053
4	Benzyl alcohol	1028	1031	1.571 ± 0.162	2.133 ± 0.201	6.321 ± 0.096	2.027 ± 0.155
5	2-Octen-1-ol, (Z)-	1062	1054	0.065 ± 0.01	0.095 ± 0.003	1.093 ± 0.102	0.158 ± 0.01
6	1-Octanol	1067	1070	0.057 ± 0.008	0.116 ± 0.011	0.213 ± 0.003	0.096 ± 0.004
7	Phenylethyl Alcohol	1105	1109	0.017 ± 0.002	0.048 ± 0.001	0.025 ± 0	0.074 ± 0.005
8	1-Nonanol	1169	1171	0.038 ± 0.004	0.039 ± 0.006	0.074 ± 0	0.122 ± 0.03
9	3-Chlorobenzyl alcohol	1234		0.009 ± 0.001	0.016 ± 0.001	0.053 ± 0.003	0.012 ± 0.002
10	Geraniol	1253	1254	^c^ ND	0.018 ± 0.003	ND	0.023 ± 0.001
11	Cedrol	1615	1607	0.017 ± 0.002	0.058 ± 0.004	0.012 ± 0	0.033 ± 0.006
12	Phenol, 5-(1,5-dimethyl-4-hexenyl)-2-methyl-, (R)-	1749		0.02 ± 0.002	0.043 ± 0.004	0.005 ± 0	0.048 ± 0.005
Aldehydes							
13	Hexanal	782	780	0.795 ± 0.116	0.673 ± 0.064	1.895 ± 0.054	0.815 ± 0.026
14	2-Hexenal, (E)-	836	856	0.015 ± 0.002	0.025 ± 0	0.035 ± 0.001	0.023 ± 0.001
15	Heptanal	893	902	0.038 ± 0.007	0.04 ± 0.002	0.047 ± 0.003	0.032 ± 0.002
16	Benzaldehyde	953	960	3.932 ± 0.473	5.642 ± 0.194	6.616 ± 0.113	5.784 ± 0.433
17	Octanal	999	1005	0.085 ± 0.018	0.096 ± 0.021	0.104 ± 0.002	0.084 ± 0.006
18	Benzeneacetaldehyde	1036	1046	0.386 ± 0.037	0.577 ± 0.043	0.146 ± 0.001	0.463 ± 0.005
19	2-Octenal, (E)-	1052	1055	0.267 ± 0.025	0.373 ± 0.017	0.524 ± 0.012	0.441 ± 0.015
20	Nonanal	1102	1108	0.474 ± 0.118	0.485 ± 0.208	0.327 ± 0.001	0.318 ± 0.027
21	Benzaldehyde, 3-chloro-	1122		0.025 ± 0.003	0.038 ± 0.003	0.012 ± 0	0.047 ± 0.002
22	2-Phenylpropenal	1147		0.022 ± 0.002	0.05 ± 0.005	ND	0.041 ± 0.003
23	2-Nonenal, (E)-	1155	1162	0.155 ± 0.01	0.143 ± 0.007	0.195 ± 0.005	0.204 ± 0.023
24	Decanal	1204	1208	0.091 ± 0.02	0.104 ± 0.011	0.107 ± 0.001	0.079 ± 0.003
25	Benzaldehyde, 3,5-dimethyl-	1207		0.389 ± 0.038	0.264 ± 0.059	0.891 ± 0.003	0.212 ± 0.012
26	2,4-Nonadienal, (E,E)-	1212	1218	0.121 ± 0.009	0.111 ± 0.008	0.402 ± 0	0.166 ± 0.007
27	Benzeneacetaldehyde, .alpha.-ethylidene-	1268	1276	0.054 ± 0.004	0.072 ± 0.004	0.058 ± 0.001	0.092 ± 0.006
28	Cinnamaldehyde, (E)-	1272	1272	0.014 ± 0.001	0.021 ± 0.001	0.015 ± 0	0.025 ± 0.001
29	Undecanal	1307	1306	0.018 ± 0.002	0.019 ± 0.002	0.024 ± 0.001	0.018 ± 0.001
30	2,4-Decadienal, (E,E)-	1317	1313	0.041 ± 0.004	0.045 ± 0.001	0.183 ± 0.002	0.059 ± 0.002
31	Dodecanal	1408	1408	0.015 ± 0.002	0.014 ± 0.001	0.032 ± 0.001	0.015 ± 0
32	2-Octenal, 2-butyl-	1373		ND	ND	0.076 ± 0.001	0.017 ± 0.001
Acids							
33	Benzoic acid	1164	1163	ND	0.096 ± 0.015	0.098 ± 0	0.087 ± 0.007
34	Tetradecanoic acid	1756	1760	0.009 ± 0	0.033 ± 0.002	0.012 ± 0	ND
Esters							
35	Benzoic acid, methyl ester	1088	1095	0.016 ± 0.001	0.026 ± 0.002	0.029 ± 0.002	0.022 ± 0
36	Acetic acid, 2-ethylhexyl ester	1145		ND	ND	0.045 ± 0	ND
37	Methyl salicylate	1184	1187	ND	0.011 ± 0.001	0.015 ± 0	0.011 ± 0.001
38	Octanoic acid, ethyl ester	1194	1197	0.014 ± 0.002	0.014 ± 0.001	0.037 ± 0.001	0.018 ± 0.001
39	Decanoic acid, methyl ester	1327	1326	0.006 ± 0	0.005 ± 0	0.008 ± 0	0.005 ± 0
40	Dodecanoic acid, methyl ester	1523	1526	0.02 ± 0.002	ND	0.033 ± 0	0.02 ± 0.001
41	Methyl tetradecanoate	1723	1723	0.036 ± 0.005	0.051 ± 0.003	0.047 ± 0.002	0.041 ± 0.004
42	Tetradecanoic acid, ethyl ester	1791	1794	ND	ND	0.016 ± 0	ND
43	2(3H)-Furanone, dihydro-5-(2-octenyl)-, (Z)-	1656		ND	ND	0.057 ± 0	ND
44	Pentadecanoic acid, methyl ester	1823		0.02 ± 0.003	0.044 ± 0.004	0.019 ± 0	0.049 ± 0.011
45	Hexadecanoic acid, methyl ester	1924	1922	0.114 ± 0.017	0.133 ± 0.031	0.104 ± 0.01	0.096 ± 0.024
46	Dibutyl phthalate	1956	1954	0.031 ± 0.006	0.063 ± 0.005	0.055 ± 0.001	0.049 ± 0.005
47	Hexadecanoic acid, ethyl ester	1992	1990	0.005 ± 0.001	0.011 ± 0	0.015 ± 0.001	0.013 ± 0.002
48	9,12-Octadecadienoic acid (Z,Z)-, methyl ester	2093	2097	0.26 ± 0.029	0.111 ± 0.032	0.278 ± 0.031	0.091 ± 0.026
Ketones							
49	1-Octen-3-one	969	981	0.072 ± 0.014	0.079 ± 0.013	0.147 ± 0.005	0.096 ± 0.013
50	3-Octen-2-one	1032		0.11 ± 0.013	0.08 ± 0.007	0.218 ± 0.011	0.1 ± 0.006
51	Acetophenone	1056	1064	0.025 ± 0.004	0.035 ± 0.002	0.023 ± 0.002	0.052 ± 0.002
52	3-Nonen-2-one	1134	1136	ND	0.02 ± 0.002	0.041 ± 0.001	0.026 ± 0.001
53	1,2-Propanedione, 1-phenyl-	1159		0.041 ± 0.01	0.077 ± 0.007	0.07 ± 0.001	0.157 ± 0.003
54	2-Undecanone	1293	1292	0.172 ± 0.008	0.159 ± 0.008	0.583 ± 0.004	0.196 ± 0.009
55	2-Butanone, 1-(1,3-benzodioxol-5-yl)-	1391		ND	0.018 ± 0.001	0.032 ± 0.002	0.016 ± 0.001
56	5,9-Undecadien-2-one, 6,10-dimethyl-, (E)-	1448	1444	0.046 ± 0.005	0.065 ± 0.006	0.1 ± 0.001	0.068 ± 0.003
57	5,9,13-Pentadecatrien-2-one, 6,10,14-trimethyl-	1912		0.011 ± 0.001	0.033 ± 0.006	0.024 ± 0.001	0.031 ± 0.005
Hydrocarbons							
58	Styrene	880	886	0.014 ± 0.001	0.014 ± 0.004	0.017 ± 0	0.011 ± 0
59	Benzene, 2-ethyl-1,4-dimethyl-	1077	1072	0.034 ± 0.005	0.023 ± 0.001	0.043 ± 0.004	0.021 ± 0.001
60	Benzene, 1,2,4,5-tetramethyl-	1112	1130	0.106 ± 0.01	0.047 ± 0.004	0.115 ± 0.011	0.048 ± 0.004
61	Benzene, 1-ethenyl-4-ethyl-	1130		0.04 ± 0.005	0.017 ± 0.002	0.04 ± 0.004	0.017 ± 0.001
62	Naphthalene, 1,2,3,4-tetrahydro-	1151	1158	0.009 ± 0.001	ND	0.016 ± 0.001	ND
63	Naphthalene	1174	1179	0.236 ± 0.026	0.148 ± 0.013	0.386 ± 0.01	0.148 ± 0.013
64	Benzene, 1-ethyl-3-(1-methylethyl)-	1189		0.033 ± 0.004	0.019 ± 0.002	0.047 ± 0.002	0.017 ± 0.001
65	Dodecane	1199	1200	0.033 ± 0.003	0.024 ± 0.002	0.057 ± 0.003	0.029 ± 0.003
66	Benzene, pentamethyl-	1276		0.015 ± 0.001	0.008 ± 0.001	0.056 ± 0.001	0.024 ± 0
67	Naphthalene, 2-methyl-	1289	1291	0.027 ± 0.003	0.026 ± 0.001	0.056 ± 0.001	0.024 ± 0.001
68	Naphthalene, 1-methyl-	1299	1308	0.015 ± 0.002	0.02 ± 0.002	0.031 ± 0.001	0.021 ± 0.001
69	.alpha.-Cubebene	1349	1352	ND	ND	0.016 ± 0	ND
70	Tetradecane	1400	1400	0.02 ± 0.001	0.028 ± 0.005	0.039 ± 0.001	0.024 ± 0.001
71	1H-3a,7-Methanoazulene, 2,3,4,7,8,8a-hexahydro-3,6,8,8-tetramethyl-, [3R-(3.alpha.,3a.beta.,7.beta.,8a.alpha.)]-	1412	1409	0.016 ± 0.001	0.025 ± 0.001	0.032 ± 0.001	0.018 ± 0.001
72	Pentadecane	1500	1500	0.045 ± 0.003	0.062 ± 0.008	0.081 ± 0.006	0.056 ± 0.006
73	Hexadecane	1600	1600	0.018 ± 0.001	0.033 ± 0.001	0.023 ± 0.001	0.027 ± 0.003
74	Heptadecane	1700	1700	0.021 ± 0.001	0.079 ± 0.018	0.032 ± 0.002	0.047 ± 0.003
75	Octadecane	1800	1800	0.009 ± 0.003	0.052 ± 0.002	0.009 ± 0	0.051 ± 0.007
76	Hexadecane, 2,6,10,14-tetramethyl-	1807	1806	0.007 ± 0	0.052 ± 0.003	0.008 ± 0.001	0.058 ± 0.005
Others							
77	Pyrazine, 2,6-dimethyl-	904	912	ND	ND	0.019 ± 0.001	0.009 ± 0.001
78	Furan, 2-pentyl-	984	994	0.173 ± 0.024	0.188 ± 0.019	0.385 ± 0.004	0.188 ± 0.01
79	Pyrazine, 3-ethyl-2,5-dimethyl-	1069	1074	ND	ND	ND	0.027 ± 0.002
80	Estragole	1286	1193	0.002 ± 0	0.017 ± 0	0.037 ± 0.001	0.016 ± 0.001
81	Benzene, 2,4-diisocyanato-1-methyl-	1352		0.008 ± 0.001	0.009 ± 0.001	0.018 ± 0.001	0.011 ± 0.001
82	2,5-Cyclohexadiene-1,4-dione, 2,6-bis(1,1-dimethylethyl)-	1461		0.03 ± 0.004	0.037 ± 0.001	0.015 ± 0	0.031 ± 0.001
83	2,4-Di-tert-butylphenol	1504	1519	0.021 ± 0.001	0.024 ± 0.003	0.043 ± 0.001	0.022 ± 0.001

^a^ Results were expressed as means ± SD (*n* = 3). Means with different letters within a row was significantly different (*p* < 0.05), the content of volatiles was the mean of relative content to that of the internal standard (Ethyl decanoate). ANOVA and Duncan test was used to analyse the significant difference between samples from different substrates. ^b^ RIs were determined using n-alkanes C7-C25 as external references. ^c^ ND: not detected.

## Data Availability

The data used to support the findings of this study can be made available by the corresponding author upon request.

## Supplementary Materials

The following supporting information can be downloaded at: https://www.mdpi.com/article/10.3390/foods12132440/s1, Table S1: Chemical composition of raw materials and the substrate formulates used in the *A. bisporus* cultivation trial. Table S2: Change of C and N content during fermentation of culture materials.
