# Supplementary material for: Cultivation and Nutritional Evaluation of Agaricus bisporus with Tea Residue as Culture Medium
Source: Foods. 2023 Jun 21;12(13):2440. doi: 10.3390/foods12132440 (PMC10341170; doi:10.3390/foods12132440)
Supplement: Supplementary file 1 [file foods-12-02440-s001.zip › foods-2408436-supplementary.pdf]

Table S1. Chemical composition of raw materials and the substrate formulates used in the *A. bisporus* cultivation trial

Raw materials	Total C and N contents			Substrate formulas (%)			
	C %[a]	N %[b]	C/N[c]	CK	T1	T2	T3
Wheat straw	42.66	0.55	77.56	70	70	60	42.5
Chicken manure	35.83	6.93	5.17	18	18	18	18
Soybean meal	45.20	4.54	10.00	10	0	0	0
Tea residue	50.40	5.91	8.52	0	10	20	37.5
Gypsum	--	--	--	1	1	1	1
Lime	--	--	--	1	1	1	1

[a] C %, Organic carbon percentage; [b] N %, total nitrogen percentage; [c] C/N, the ratio of the organic carbon content (%) to the total nitrogen content (%) in dried compost.

Table S2 Change of C and N content during fermentation of culture materials

Formulas	Unfermented substrate			Fermented substrate			Residual rate	
	C %[a]	N %[b]	C/N[c]	C %[a]	N %[b]	C/N[c]	C %[a]	N %[b]
CK	34.19	1.80	19.02	30.85	1.59	19.42	90.22	88.48
T1	36.15	1.93	18.69	32.12	1.65	19.47	88.85	85.31
T2	36.92	2.47	14.95	32.45	1.75	18.54	87.89	70.85
T3	38.28	3.41	11.23	33.26	1.98	16.8	86.89	58.06

[a] C %, Organic carbon percentage; [b] N %, total nitrogen percentage; [c] C/N, the ratio of the organic carbon content (%) to the total nitrogen content (%) in dried compost.
